# Spotlight on the Effect of Electrolyte Composition on the Potential of Maximum Entropy: Supporting Electrolytes Are Not Always Inert

**DOI:** 10.1002/chem.202101537

**Published:** 2021-06-09

**Authors:** Xing Ding, Batyr Garlyyev, Sebastian A. Watzele, Theophilus Kobina Sarpey, Aliaksandr S. Bandarenka

**Affiliations:** ^1^ Physics of Energy Conversion and Storage Technical University of Munich James-Franck-Strasse 1 85748 Garching Germany; ^2^ Catalysis Research Center TUM Technical University of Munich Ernst-Otto-Fischer-Strasse 1 85748 Garching Germany

**Keywords:** electrocatalysis, electrolyte influence, gold electrodes, laser-induced current transient, potential of maximum entropy

## Abstract

The influence of electrolyte pH, the presence of alkali metal cations (Na^+^, K^+^), and the presence of O_2_ on the interfacial water structure of polycrystalline gold electrodes has been experimentally studied in detail. The potential of maximum entropy (PME) was determined by the laser‐induced current transient (LICT) technique. Our results demonstrate that increasing the electrolyte pH and introducing O_2_ shift the PME to more positive potentials. Interestingly, the PME exhibits a higher sensitivity to the pH change in the presence of K^+^ than Na^+^. Altering the pH of the K_2_SO_4_ solution from 4 to 6 can cause a drastic shift in the PME. These findings reveal that, for example, K_2_SO_4_ and Na_2_SO_4_ cannot be considered as equal supporting electrolytes: it is not a viable assumption. This can likely be extrapolated to other common “inert” supporting electrolytes. Beyond this, knowledge about the near‐ideal electrolyte composition can be used to optimize electrochemical devices such as electrolyzers, fuel cells, batteries, and supercapacitors.

## Graphical abstract

**Gold standards**: By determining the potential of maximum entropy (PME) with the laser‐induced current transient technique, the influence of the electrolyte composition on polycrystalline gold has been systematically investigated. Increasing the electrolyte pH and purging O_2_ shift the PME to more positive potentials. The electric double‐layer structure for Au_pc_ in solutions containing Na^+^ and K^+^ ions is vastly different when electrolyte pH reaches six or higher. This method enables another approach for the interpretation of interfacial electrocatalytic processes.

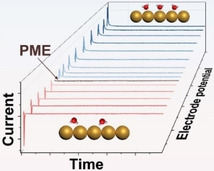

Understanding the properties of the electric double layer (EDL) formed between the electrodes and electrolytes is significant for the rational design of energy conversion and storage systems.^[1]^ One of the critical interfacial properties is the net orientation of solvent (e. g., water) molecules at the electrode/electrolyte interface.^[2]^ The interfacial water structure, determined by the charge separation at the interface, is influenced by electrode potential, pH of the electrolyte, and its composition.^[3]^ A parameter associated with the net orientation of water molecules is the so‐called potential of maximum entropy (PME), which is defined as the potential at which the entropy of double‐layer formation reaches its maximum.^[4]^ Knowledge of the PME is critical for assessing the stiffness of the water layer at the interface and, therefore, can be considered as the main reason for energy barriers that hinder mass and charge transfer through the interface.^[2a]^ At potentials remote from the PME, the water layer at the interface is relatively rigid, which hinders the charge transfer. Inversely, at potentials close to the PME, the interfacial water has the highest disorder and can reorient more freely, enabling easier charge transfer.

The PME can be determined by the laser‐induced current transient (LICT) technique.^[5]^ This methodology is based on the sudden temperature jump effect caused by nanosecond laser pulses. The current relaxation curves are measured at the potentiostatic conditions after laser probing (Scheme 1).[^2b^, ^5^] The response from the system after the laser pulse shows the information about the net orientation of water (solvent) molecules at the interface. Therefore, the potential at which the current transient changes its sign corresponds to the state with the maximum disorder, the PME.^[4]^ This in situ method has been used for studying the interfacial fundamentals of electrodes, such as the investigation of the electric double layer on Au(111) and Pt(111),^[6]^ the kinetics of the electrochemical process on Pt electrodes,^[7]^ and the correlation between the interfacial water network and the activity of Pt electrodes.[^2a^, ^8^]

**Scheme 1:**
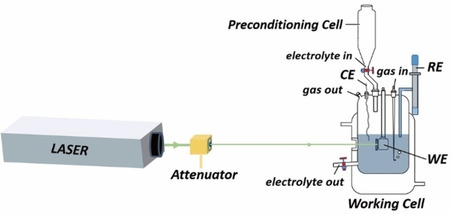
Scheme of the experimental setup used to perform the LICT measurements. WE, RE and CE correspond to working, reference, and counter electrode, respectively. More details about the LICT setup can be found in the Supporting Information.

Recent studies showed that in many cases, the electrolyte composition affects the rate of catalyzed reactions.^[9]^ Particularly interesting is the influence of the electrolyte pH and the nature of alkali metal cations present. In all of these cases, the ions interact relatively strongly with the surface affecting the electrochemical processes. For instance, it was shown that the oxygen evolution both in acidic and alkaline media depends on the nature of those “spectator species”.^[10]^ Likewise, the hydrogen evolution reaction in alkaline media was found to be largely influenced by the electrolyte composition.^[11]^ However, it is difficult for electrochemists to experimentally establish how the electrolyte compositions affect the electrode/electrolyte interfacial structure and eventually control electrocatalytic activity.

Here, we determine how the PME changes with electrolyte composition for polycrystalline gold (Au_pc_) electrodes. The investigations were performed in Ar‐ and O_2_‐saturated 0.5 M Na_2_SO_4_ and K_2_SO_4_ solutions of different pH with the LICT technique. Na_2_SO_4_ and K_2_SO_4_ were chosen because they are often used as supporting electrolytes in the industry.^[12]^ Note, the SO_4_^2−^ anion with very high hydration energy was selected to minimize the possible disruption of the anion competition. Interestingly, the PME shifts towards more positive potentials with increasing the pH values for Au_pc_ electrodes in either Ar‐saturated or O_2_‐saturated 0.5 M Na_2_SO_4_ and K_2_SO_4_ electrolytes. However, the PME exhibits a higher sensitivity to the pH in the presence of K^+^ in comparison to Na^+^. Especially, the PMEs of Au_pc_ in Na_2_SO_4_ and K_2_SO_4_ solutions are significantly different at pH 6 or higher, which indicates the drastic variation of the EDL structure in the presence of these two electrolytes. Moreover, the PME values measured in O_2_‐saturated solutions are more positive than those measured in Ar‐saturated electrolytes.

Initially, the surface quality of the Au electrode was analyzed with cyclic voltammograms (CVs) in Ar‐saturated 0.5 M Na_2_SO_4_ and K_2_SO_4_ solutions at different pHs. The CVs are shown in Figure 1. For both Na^+^‐ and K^+^‐containing electrolytes at pH 2, CVs of Au_pc_ are comparable with the measurements recorded in 0.1 M H_2_SO_4_ (see Figure S1 in the Supporting Information). The typical three anodic peaks observed between about 1.3 and 1.6 V versus the reversible hydrogen electrode (RHE) and a sharp reduction peak at about 1.1 V versus RHE can be observed.^[13]^ However, Figure 1 shows that the change in the pH strongly influences the oxidation and reduction processes on Au_pc_ surface in the Na_2_SO_4_ and K_2_SO_4_ solutions. Especially, when increasing the pH of the solution to 6, two broad reduction peaks are observed for Na^+^‐containing electrolytes and three peaks in the presence of K^+^ within the investigated potential range (Figure S2). This could be because, in a nearly neutral pH solution, the local protons at the interface have a low concentration. Hence, the local pH at the electrode/electrolyte interface can be easily changed during the oxidation and reduction processes on Au_pc_ surface. During the reduction process, the protons at the interface can be quickly consumed, which could result in the reduction of Au oxide at lower potentials.^[14]^ When the pH is kept constant at pH 6, one can observe the influence of the nature of the alkali metal cation, as shown in Figure S2. It seems that K^+^ cations promote the oxidation process of Au, which is observed from the increased peak areas compared to Na^+^ in Figure S2.

**Figure 1:**
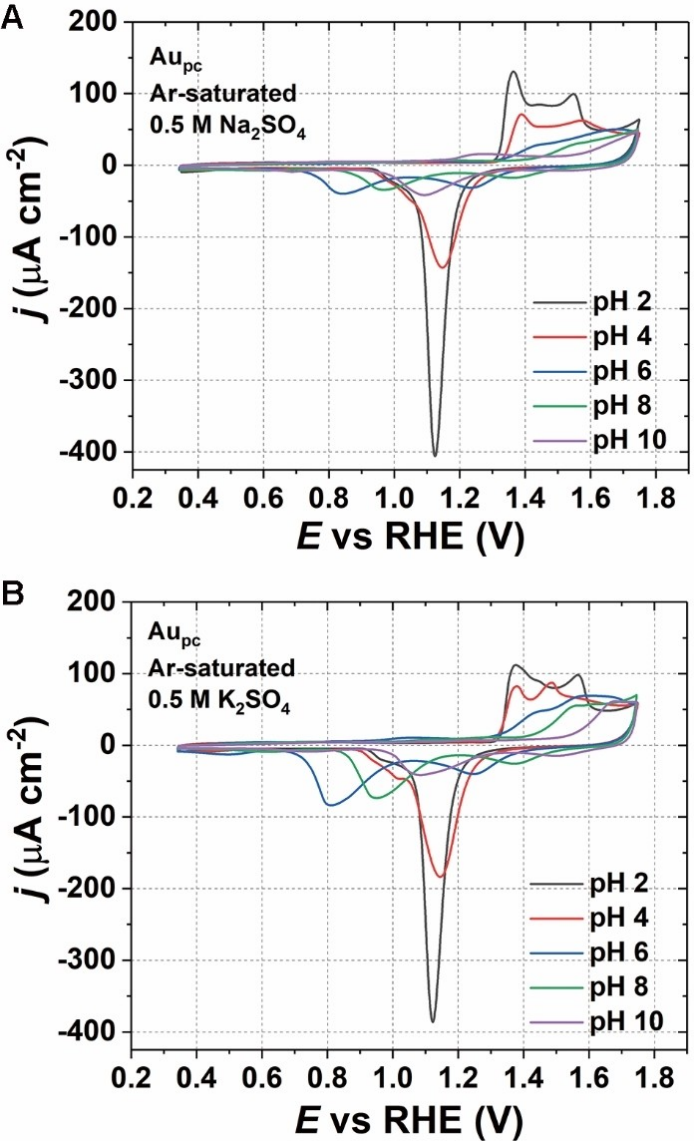
Typical cyclic voltammograms of Au_pc_ in 0.5 M Ar‐saturated A) Na_2_SO_4_ and B) K_2_SO_4_ electrolytes of different pH values (2, 4, 6, 8, and 10). Scan rate: 50 mV s^−1^.

To further investigate the influence of pH and cations on Au_pc_, the LICT measurements were performed to determine the position of the PME. The analysis was conducted in both Ar‐ and O_2_‐saturated electrolytes. Figure 2 shows the current transients corresponding to Au_pc_ in Ar‐ and O_2_‐saturated 0.5 M Na_2_SO_4_ solutions (pH 2). Since the sign of the charge of the electrode surface coincides with the current transient, a positive surface charge leads to a positive current transient and vice versa, as illustrated in Figure 2 A and C. Alternatively, the extreme values of the current transients (*i*_Xtrm_) can be plotted as a function of the applied potential to find the PME, as indicated in Figure 2 B and D. The potential at which the current transient changes its sign corresponds to the PME. Thus, the PME values for Au_pc_ are 0.19 and 0.35 V in the Ar‐ and O_2_‐saturated 0.5 M Na_2_SO_4_ solutions (pH 2), respectively.

**Figure 2:**
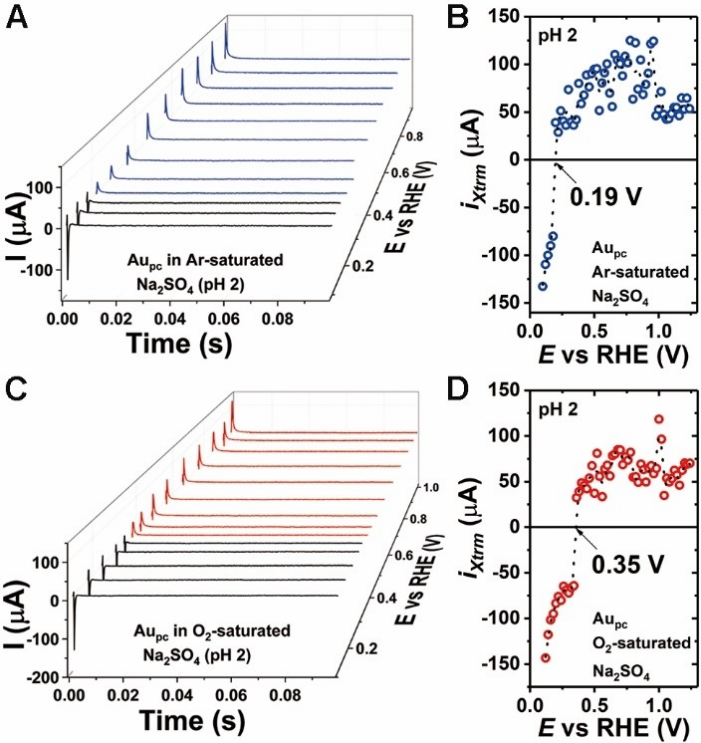
3D plots of current transients observed after laser pulse with the LICT technique for Au_pc_ in 0.5 M A) Ar‐ and C) O_2_‐saturated Na_2_SO_4_ solutions (pH 2). The negative current transients in the 3D plots are marked in black. B), D) The corresponding extreme values of the current transients (*i*_Xtrm_) as a function of the working electrode potential used to find the PME.

The PME shifts towards more positive potentials due to the pH increase for both Ar‐ and O_2_‐saturated Na_2_SO_4_ solutions (Figure 3), which means the PME depends on the pH. However, it should be noted that the pH dependence of the PME also involves the effect of adsorption processes on the free charge distribution at the interface.[^4^, ^15^] Because the PME is believed to be independent of the electrolyte pH on the standard hydrogen electrode (SHE) scale when the PME lies within the potential region without adsorption effect.^[16]^ However, it does not necessarily follow this rule at potentials where adsorption processes influence the free charge.^[15]^ In the case of Ar‐saturated Na_2_SO_4_ (Figure 3 A–D), the potentials at which the current transient changes its sign are 0.25, 0.34, 0.58, and 0.88 V for pHs 4, 6, 8, and 10, respectively. While for Au_pc_ in O_2_‐saturated Na_2_SO_4_ solutions (Figure 3 E–H), the locations of the PME are 0.39, 0.46, 0.66, and 0.98 V for pHs 4, 6, 8, and 10, respectively. One can notice that the PME values measured in O_2_‐saturated Na_2_SO_4_ solutions are more positive than the values measured in Ar‐saturated solutions for all investigated pH ranges. This is because protons can be consumed at the electrode surface by dissolved O_2_ during the laser measurements, which leads to a change of the local pH at the interface between electrode and electrolyte. Consequently, this shifts the location of the PME. Taking this result into consideration, it would also be possible to estimate the local pH at the interface by determining the PME.

**Figure 3:**
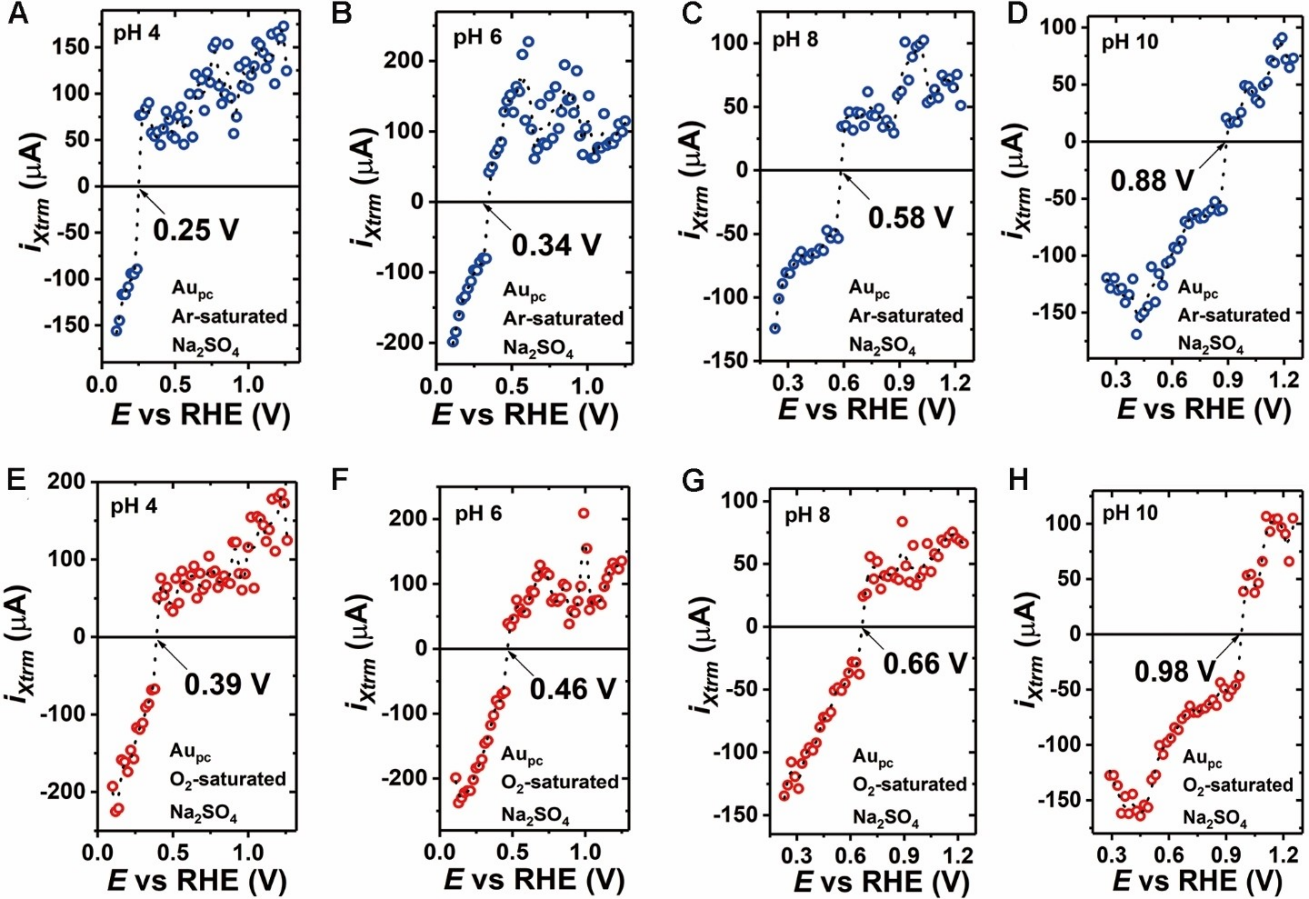
A)–D) Determination of the PME values of the Au_pc_ electrode in 0.5 M Ar‐saturated Na_2_SO_4_ electrolytes at different pH values (4, 6, 8, and 10). E)–H) In O_2_‐saturated Na_2_SO_4_ solutions, the PME increases compared to the case of Ar‐saturated electrolytes.

Similar trends are observed for Au_pc_ at different pHs of 0.5 M K_2_SO_4_ solutions, as shown in Figure S3. Namely, increasing pH and introducing O_2_ can move the PME to more positive potentials with the exception of the case of Au_pc_ in O_2_‐saturated K_2_SO_4_ from pH 6 to 8. For Au_pc_ in Ar‐saturated K_2_SO_4_ solutions (Figure S3 A–E), the PME values are 0.13, 0.27, 1.26, 1.30, and 1.43 V for pHs 2, 4, 6, 8, and 10, respectively. While the PMEs are shifted to 0.27, 0.37, 1.40, 1.38, and 1.49 V in the case of O_2_‐saturated K_2_SO_4_ solutions for pHs 2, 4, 6, 8, and 10, respectively (Figure S3 F–J). Surprisingly, for Au_pc_ in K_2_SO_4_ solutions at pH 6, 8, and 10, the PMEs are more positive than the thermodynamic equilibrium potential of the oxygen reduction reaction (1.23 V vs. RHE) but are still moved in the presence of O_2_. This could be because the water structure at the electrode/electrolyte interface can be influenced not only by the dissolved O_2_ but also by adsorbed H^+^, OH^−^, and SO_4_^2−^ on the electrode surface.

When comparing the influence of Na^+^ and K^+^ on Au_pc_, the PME exhibits a higher sensitivity to the pH in the presence of K^+^ than Na^+^ (Figure 4). The pH effect on the PME increases slightly in alkaline solutions in the presence of Na^+^. However, the PME can be drastically changed from pH 4 to 6 for Au_pc_ in K^+^‐containing electrolytes, which means that small alterations of pH within this pH range can alter the EDL structure drastically. One possible reason is that the specific adsorption of *OH (*  represents the adsorbed species) and sulfate adsorption lead to the phase transition and interfacial water rearrangements in the double layer^[17]^ and then shifts the location of the PME. When the electrolyte pH is 6 or higher, the difference of the PME value for Au_pc_ in Na_2_SO_4_ and K_2_SO_4_ becomes large (ca. 0.92 V at pH 6). In this case, Na_2_SO_4_ and K_2_SO_4_ cannot be considered as equal supporting electrolytes. Even at the same conditions, the EDL structure in the presence of these two cations differs drastically. Accordingly, the performance of the electrocatalytic reactions can be significantly changed due to the electrolyte. Combining the CVs in Figure 1, one can deduce that a strong phase transition happens for Au_pc_ in the presence of K^+^. Moreover, the difference in the PME values in the presence of K^+^ and Na^+^ could also be due to the influence of these two cations on the local pH.

**Figure 4:**
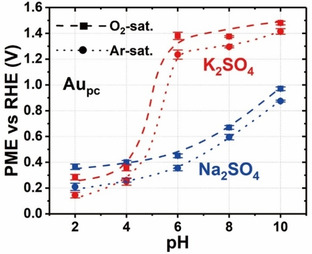
PME values for Au_pc_ in Ar‐ and O_2_‐saturated 0.5 M Na_2_SO_4_ (blue) and K_2_SO_4_ (red) solutions depicted as a function of the electrolyte pH. The dashed (O_2_‐saturated electrolytes) and dotted lines (Ar‐saturated electrolytes) are a guide for the eye.

To further check the cation effect, the LICT experiments were performed in a mixed solution (60 mL 0.5 M Na_2_SO_4_ and 60 mL 0.5 M K_2_SO_4_) at pH 8. As shown in Figure S4, the PMEs are 0.96 and 1.08 V in the Ar‐ and O_2_‐saturated mixed solutions, respectively. Interestingly, the values of the PME for Au_pc_ in this solution are located between the PMEs of Au_pc_ in Na_2_SO_4_ and K_2_SO_4_ solutions at the same pH value. This supports our hypothesis on the cation effect on the interfacial structure of Au_pc_.

In conclusion, we have determined the PME by using the LICT technique to investigate the influence of alkali metal cations, O_2_, and pH on the interfacial structure of Au_pc_. The presented results demonstrated that an increase in the pH and introduction of O_2_ could shift the PME towards more positive potentials in either Na_2_SO_4_ or K_2_SO_4_ solution owing to the change in local pH at the interface. When the pH reaches six or higher, the EDL structure for Au_pc_ in Na^+^‐ and K^+^‐containing electrolytes is significantly different because of the large difference in the PME location. Thus, Na_2_SO_4_ and K_2_SO_4_ cannot be considered as equal supporting electrolytes in this case. Our findings reveal that the electrolyte composition, even if it is considered as a supporting electrolyte, affects the EDL structure, changes the location of the PME, and influences the electrode processes. Therefore, the determination of the PME by the LICT method can be a particularly valuable method to evaluate and investigate the influence of the electrolyte composition on the electrochemical processes happening at the electrode/electrolyte interface. We believe this powerful tool can help improve the fundamental understanding of electrolyte effects, which is a prerequisite for rational electrolyte engineering.

## Conflict of interest

The authors declare no conflict of interest.

## Supporting information

As a service to our authors and readers, this journal provides supporting information supplied by the authors. Such materials are peer reviewed and may be re‐organized for online delivery, but are not copy‐edited or typeset. Technical support issues arising from supporting information (other than missing files) should be addressed to the authors.

Supplementary
