# Supplementary material for: Spotlight on the Effect of Electrolyte Composition on the Potential of Maximum Entropy: Supporting Electrolytes Are Not Always Inert
Source: Chemistry. 2021 Jun 9;27(39):10016–20. doi: 10.1002/chem.202101537 (PMC8361723; doi:10.1002/chem.202101537)
Supplement: Supplementary file 1 — Supplementary [file CHEM-27-10016-s001.pdf]

Chemistry–A European Journal

Supporting Information

Spotlight on the Effect of Electrolyte Composition on the Potential of Maximum Entropy: Supporting Electrolytes Are Not Always Inert

Xing Ding, Batyr Garlyyev, Sebastian A. Watzele, Theophilus Kobina Sarpey, and Aliaksandr S. Bandarenka*

Table of Contents

Experimental Section 2

Supporting Figures 3

Figure S1. The CV collected after cleaning for Au_{pc} in 0.1 M H_2SO_4 solution 3

Figure S2. CVs of Au_{pc} in Na_2SO_4 and K_2SO_4 at pH=6. 3

Figure S3. Determination of the PME for Au_{pc} in different pHs of K_2SO_4 electrolytes 4

Figure S4. Determination of the PME of Au_{pc} in electrolyte of 60 mL 0.5 M Na_2SO_4 and 60 mL 0.5 M K_2SO_4 at pH 8 4

Figure S5. Scheme of the electrochemical cell for the LICT experiments. 5

Figure S6. Picture of the laser experimental setup 5

References 6

Experimental Section

Cell preparation. The laser-induced current transient (LICT) measurements were performed in a custom-built electrochemical glass cell using a three-electrode configuration (**Figure S5**). The cell consists of two parts: the preconditioning compartment and the working compartment. The preconditioning compartment was used for the preparation of electrolytes with purging gases. The main cell has a flat glass window on one side which allows the laser beam to directly reach the working electrode surface (through the electrolyte). Additionally, the cell has a temperation mantle which allows temperature control of the investigated electrolytes by a water thermostat (Julabo, Germany). Before the measurements, all cell parts were carefully cleaned with a piranha solution, 3:1 mixture of H_2SO_4 (96% Suprapur, Merck, Germany) and H_2O_2 (30% Suprapur, Merck, Germany), and then rinsed with boiling ultrapure water several times. A mercury-mercury sulfate reference electrode and a Pt wire (counter electrode) were used for the electrochemical experiments. All potentials shown in this paper are converted to the reversible hydrogen electrode (RHE) scale.

Working electrode preparation. AT-cut polycrystalline Au quartz crystal wafer electrodes (Stanford Research Systems, USA) with a surface area of 1.37 cm^2 were used for the LICT measurements. Before the measurements, the polycrystalline Au (Au_{pc}) electrodes were electrochemically cleaned in Ar-saturated 0.1 M H_2SO_4 solutions. The cleaning procedures were performed within the potential range of 0.40 – 2.10 V until the voltammograms were stable. To ensure the quality of the working electrode surface, the voltammograms within the potential regime of 0.50 – 1.70 V were measured (see **Figure S1**) to further compare with those results shown in the literature.[1]

Electrolyte preparation. 0.5 M Na_2SO_4 and K_2SO_4 solutions were obtained by dissolving the powders of Na_2SO_4 (99.0%, Alfa Aesar) and K_2SO_4 (99.0%, Alfa Aesar). The pH values of solutions were adjusted with either H_2SO_4 or NaOH (99.996%, Alfa Aesar) and KOH (99.98%, Alfa Aesar) using a PHHH222 portable pH meter (Omega).

LICT measurements. A Quanta-Ray INDI pulsed Nd:YAG laser (Spectra-Physics Lasers) with 532 nm wavelength was applied for the LICT measurements. The duration of the laser pulse is 5–8 ns and its repetition rate is 10 Hz. The diameter of the laser beam was around 9 mm. To avoid destroying the electrode surface, a motorized beam splitter (Newport Corp) was employed as an attenuator. Laser energy was kept at 20 mJ (ca. 32 mJ cm^{-2}) for all investigated systems. The photograph of the LICT setup is shown in **Figure S6**. The LICT measurements were carried out potentiostatically with a step of 20 mV. The current transients were recorded after each laser pulse. More information about the description of LICT experiments has been reported by our group.[2]

Supporting Figures

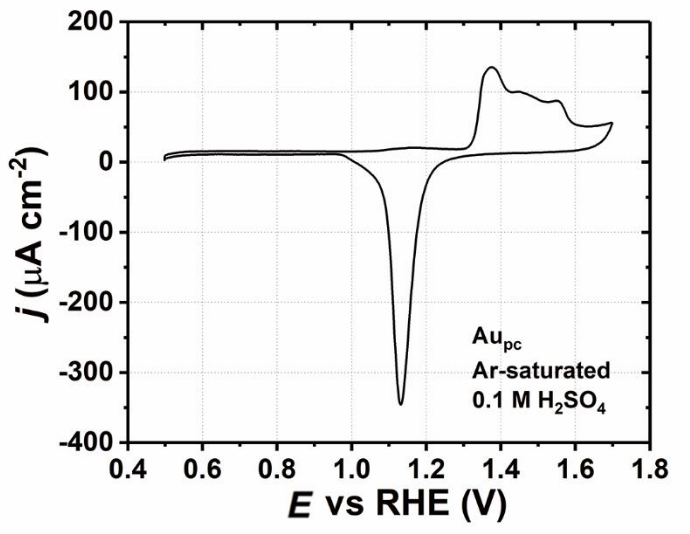

Figure S1. The stable cyclic voltammogram collected after cleaning for Au$_{pc}$ in 0.1 M H_2SO_4 solution. Scan rate: 50 mV s^{-1}.

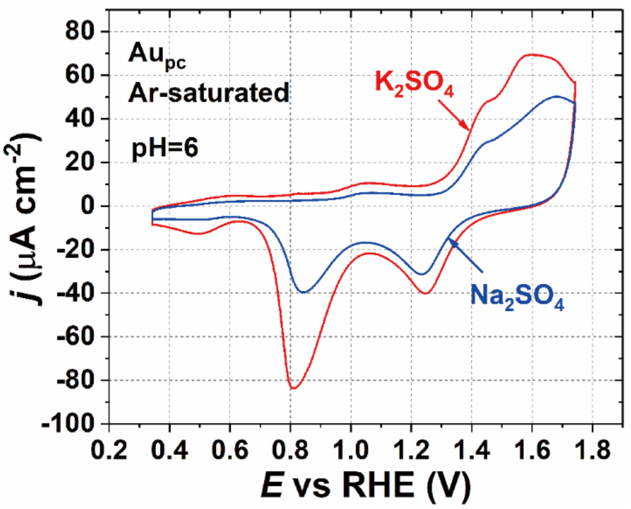

Figure S2. Cyclic voltammograms of Au$_{pc}$ in 0.5 M Ar-saturated Na_2SO_4 (blue) and K_2SO_4 (red) electrolytes at pH=6. Scan rate: 50 mV s^{-1}.

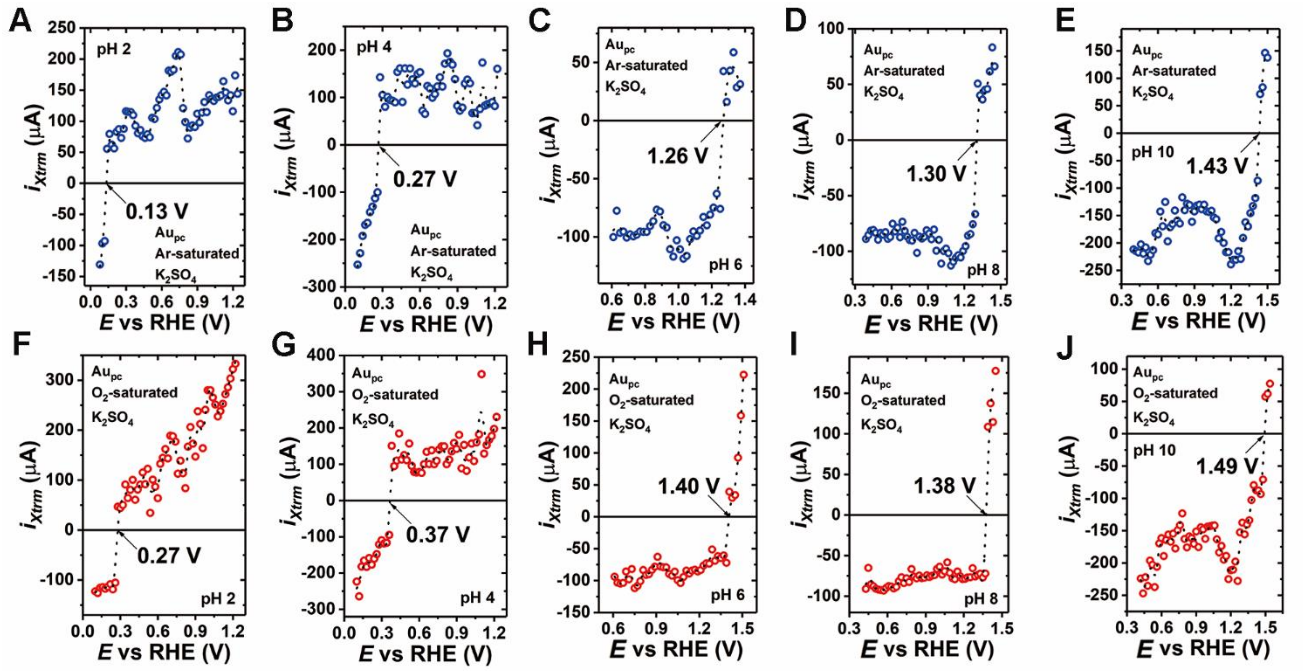

Figure S3. (A–E) Determination of the PME for Au_{pc} in different pHs (2, 4, 6, 8, and 10) of 0.5 M Ar-saturated K_2SO_4 electrolytes. A similar trend with Na_2SO_4, (F–H) the PME moves to more positive potentials for Au_{pc} in O_2-saturated K_2SO_4 electrolytes.

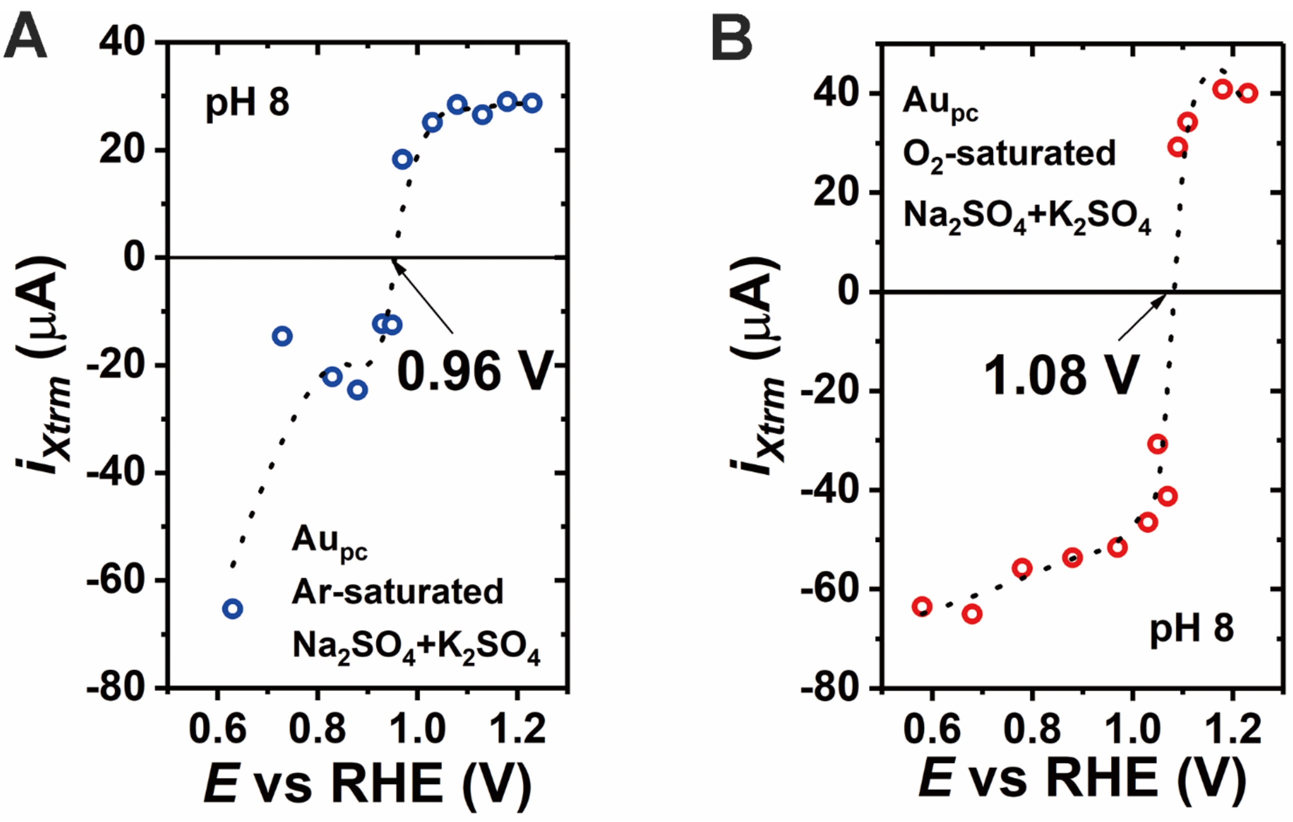

Figure S4. Determination of the PME of Au_{pc} in (A) Ar-saturated and (B) O_2-saturated mixed solution of 60 mL 0.5 M Na_2SO_4 and 60 mL 0.5 M K_2SO_4 at pH 8.

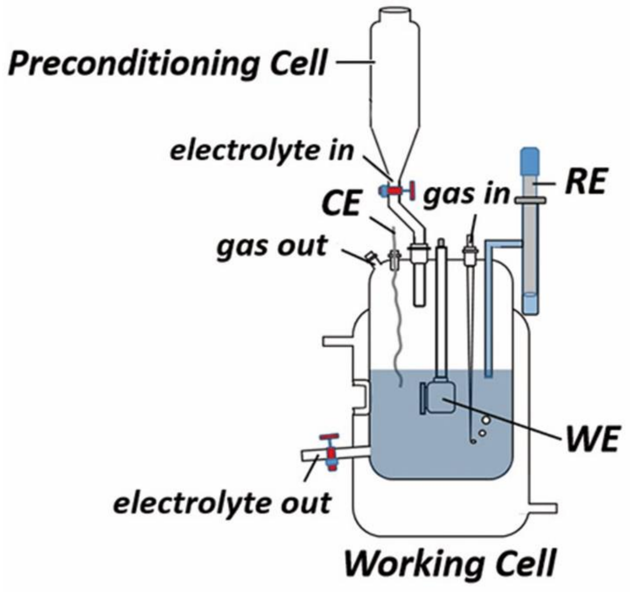

Figure S5. Scheme of the electrochemical cell for the LICT experiments. WE, RE, and CE correspond to working electrode, reference electrode, and counter electrode, respectively.

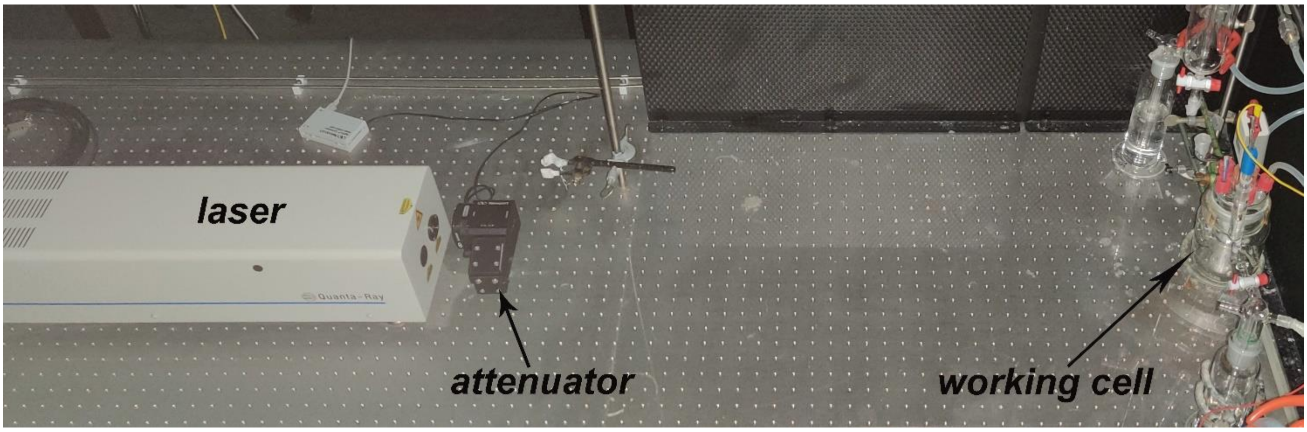

Figure S6. Picture of the laser experimental setup, which includes a Quanta-Ray INDI pulsed Nd:YAG laser head, a motorized beam splitter (attenuator), and a working cell.

References

[1] a) W. Ma, Y.-L. Ying, L.-X. Qin, Z. Gu, H. Zhou, D.-W. Li, T. C. Sutherland, H.-Y. Chen, Y.-T. Long, *Nat. Protoc.* **2013**, *8*, 439; b) A. R. Silva Olaya, B. Zandersons, G. Wittstock, *ChemElectroChem* **2020**, *7*, 3670-3678.

[2] a) D. Scieszka, C. Sohr, P. Scheibenbogen, P. Marzak, J. Yun, Y. Liang, J. Fichtner, A. S. Bandarenka, *ACS Appl. Mater. Interfaces* **2018**, *10*, 21688-21695; b) D. Scieszka, J. Yun, A. S. Bandarenka, *ACS Appl. Mater. Interfaces* **2017**, *9*, 20213-20222.
